# Insulin Resistance in Skeletal Muscle of Chronic Stroke

**DOI:** 10.3390/brainsci11010020

**Published:** 2020-12-26

**Authors:** Alice S. Ryan, Charlene Hafer-Macko, Heidi K. Ortmeyer

**Affiliations:** 1 VA Research Service, Department of Medicine, Division of Gerontology and Geriatric Medicine, University of Maryland School of Medicine, Baltimore, MD 21201, USA; heidi.ortmeyer@va.gov; 2 Baltimore VA Medical Center Geriatric Research, Education and Clinical Center (GRECC), VA Maryland Health Care System, Baltimore, MD 21201, USA; charlene.hafer-macko@va.gov

**Keywords:** muscle metabolism, glucose clamp, insulin-stimulation

## Abstract

A stroke can lead to reduced mobility affecting skeletal muscle mass and fatty infiltration which could lead to systemic insulin resistance, but this has not been examined and the mechanisms are currently unknown. The objective was to compare the effects of in vivo insulin on skeletal muscle glycogen synthase (GS) activity in paretic (P) and nonparetic (NP) skeletal muscle in chronic stroke, and to compare to nonstroke controls. Participants were mild to moderately disabled adults with chronic stroke (*n* = 30, 60 ± 8 years) and sedentary controls (*n* = 35, 62 ± 8 years). Insulin sensitivity (M) and bilateral GS activity were determined after an overnight fast and during a hyperinsulinemic-euglycemic clamp. Stroke subjects had lower aerobic capacity than controls, but M was not significantly different. Insulin-stimulated activities of GS (independent, total, fractional), as well as absolute differences (insulin minus basal) and the percent change (insulin minus basal, relative to basal) in GS activities, were all significantly lower in P versus NP muscle. Basal GS fractional activity was 3-fold higher, and the increase in GS fractional activity during the clamp was 2-fold higher in control versus P and NP muscle. Visceral fat and intermuscular fat were associated with lower M. The effect of in vivo insulin to increase GS fractional activity was associated with M in control and P muscle. A reduction in insulin action on GS in paretic muscle likely contributes to skeletal muscle-specific insulin resistance in chronic stroke.

## 1. Introduction

A person who sustains a stroke has reduced mobility, is at an increased risk of recurrent stroke with the number of strokes expected to substantially increase over the next two decades [1]. In middle-aged and older adults, a history of stroke is the sixth leading cause of disability-adjusted life years, not far behind the associated comorbidities of hypertension and diabetes [2]. Our previous findings indicate that the paretic thigh of stroke survivors has 20–24% lower muscle area and muscle volume and 17–25% higher intermuscular adipose tissue (IMAT) than the nonparetic thigh [3,4]; factors which we have shown are associated with reduced aerobic capacity and strength [4,5]. Although IMAT is associated with insulin resistance in other populations [6,7,8], this has not yet been examined in stroke survivors. We have also shown a shift to fast myosin chain phenotype [9] and higher skeletal muscle inflammation in paretic muscle compared to nonparetic muscle and controls [10] which could also lead to systemic insulin resistance in chronic stroke.

Insulin resistance is recognized as an independent risk factor for stroke and cardiovascular disease and is highly prevalent during chronic recovery from stroke. In the Uppsala Longitudinal Study of Adult Men who were free from stroke at age 70 years and followed for approximately 9 years, a one standard deviation increase in insulin sensitivity measured by a euglycemic clamp decreased the risk for subsequent stroke or transient ischemic attack (TIA) [11]. Neither the hyperinsulinemic-euglycemic clamp technique, the gold standard in the determination of insulin sensitivity, and the measurement of glycogen synthase (GS) activity, a known benchmark index of insulin action in skeletal muscle, have ever been collectively determined in stroke survivors. 

We hypothesized that stroke survivors will be insulin resistant by the glucose-clamp technique and have lower insulin-activation of GS compared to controls, and that insulin activation of GS will be more impaired in paretic compared to nonparetic skeletal muscle. The aim of this study was to determine insulin sensitivity and bilateral insulin activation of muscle GS in paretic and nonparetic skeletal muscle in chronic stroke survivors and to compare basal and insulin-stimulated GS activity to nonstroke controls. 

## 2. Materials and Methods

### 2.1. Subjects

Stroke survivors (*n* = 30, 18 men, 12 women) between 45 and 75 years and BMI between 19 and 45 kg/m^2^ (range underweight to morbid obesity) with residual hemiparetic deficits > 6 months after onset of ischemic stroke participated in the study. Participants were recruited through local advertisements, flyers, and stroke clinics at the University of Maryland School of Medicine and Baltimore VA Medical Center. All participants with stroke had mild to moderate hemiparetic gait deficits characterized by reduced stance or reduced stance with increased swing duration and had completed conventional rehabilitation therapy. All subjects lived independently in the community and were able to walk 6 min with their usual assistive device(s) without human assistance. Ambulation was the primary means of mobility for participants with stroke. Use of an ankle-foot orthosis (AFO) or assistive device was documented. Evaluations included medical history, physical examination, fasting blood profile, and screening for dementia [12] and depression [13] to ensure adequate informed consent. Participants with stroke were excluded if they had unstable angina, congestive heart failure (NYHA II), severe peripheral arterial disease, end-stage organ disease, major post-stroke depression, dementia, severe receptive aphasia, orthopedic or chronic pain conditions. Healthy control counterparts (*n* = 35, 17 men and 18 women) had the same exclusion criteria as participants with stroke and were 45–81 years of age with a BMI between 25 and 45 kg/m^2^. All stroke and healthy control participants were sedentary as defined by <20 min low intensity exercise 3x/week.

The Institutional Review Board of the University of Maryland and the Baltimore VA Research and Development Committee (HP-00041347) approved all methods and procedures. Each participant provided written informed consent.

### 2.2. VO_2_peak and Body Composition

VO_2_peak (peak oxygen consumption or aerobic capacity) was measured using a continuous treadmill test protocol with open circuit spirometry in controls [14]. In the stroke participants, a similar exercise treadmill test with open circuit spirometry was conducted to measure VO_2_ peak [15]. Height (cm) and weight (kg) were measured. Fat mass, lean tissue mass, and %body fat were determined by dual-energy X-ray absorptiometry (DXA) (GE Lunar iDXA, Madison, WI, USA). CT scans (Siemens Somatom Sensation 64 Scanner, Fairfield, CT, USA) at L_2_-L_3_ region and L_4_-L_5_ region were used to determine visceral adipose tissue area, subcutaneous adipose tissue area, and analyzed using Medical Image Processing, Analysis and Visualization, v.7.0.0. A third scan at the mid-thigh was used to quantify muscle area, total fat area, and low density lean tissue (IMAT) [16]. Muscle area and IMAT were normalized for the muscle size of the paretic and nonparetic leg by calculating a percentage of each measure relative to the muscle cross sectional area. Three stroke participants and two controls did not undergo CT scans due to scheduling difficulty.

### 2.3. Oral Glucose Tolerance Test (OGTT)

After a 12-h overnight fast, the participants had a blood draw before and at 30-min intervals for 2 h after ingestion of 75 g glucose. Samples were collected in heparinized syringes, placed in prechilled test tubes containing 1.5 mg EDTA/mL blood, centrifuged at 4 °C and stored at −80 °C until analysis. Plasma glucose concentrations were measured using the glucose oxidase method (2300 STAT Plus, YSI, Yellow Springs, OH, USA). Plasma insulin was measured in duplicate by radioimmunoassay (Millipore, St. Charles, MO, USA). The participants were defined by glucose tolerance status. 

### 2.4. Hyperinsulinemic-Euglycemic Clamps with Skeletal Muscle Biopsies and Substrate Oxidation 

All participants were weight stabilized (±2%) for at least 2 weeks prior to metabolic testing and were provided all meals as a eucaloric diet for two days before the clamp by a registered dietitian to control nutrient intake [16]. All testing was performed in the morning after a 12-h overnight fast. Whole body insulin sensitivity was measured using the hyperinsulinemic-euglycemic clamp technique [17]. Arterialized blood was obtained from a dorsal heated hand vein [18]. Blood samples were obtained every 5 and 10 min for the determination of plasma glucose and insulin levels. A 10 min priming and continuous infusion of insulin (80 mU·m^−2^·min^−1^ Humulin, Eli Lilly Co., Indianapolis, IN, USA) was performed for 180 min with a continuous infusion of 20% glucose solution starting at 10 min. Blood samples were collected in heparinized syringes, placed in prechilled test tubes containing 1.5 mg EDTA/mL of blood and centrifuged at 4 °C for plasma glucose and stored at −70 °C until analysis for plasma insulin [19]. Plasma insulin concentrations during 120–180 min of the hyperinsulinemic-euglycemic clamps was not different between stroke and controls (1214 ± 281 vs. 1190 ± 201 pmol/L). M (glucose utilization) was calculated from the amount of glucose infused after correction for glucose equivalent space (glucose space correction). Four stroke participants did not undergo the glucose clamp due to inability to obtain IV access but underwent basal muscle biopsies. Prior to the start of the clamp and 120-min during the glucose clamp, a vastus lateralis muscle biopsy was taken from each participant under local anesthesia for the measurement of GS activity. Specifically, each stroke participant had bilateral biopsies (paretic and nonparetic) for a total of four muscle biopsies. Twenty-four hour urine collections were collected the day prior to each clamp. Continuous indirect calorimetry was performed prior to and during the last 30 min of the insulin infusion by the open circuit dilution technique using a COSMED Quark RMR cart (Chicago, IL, USA) to quantitate rates of glucose oxidation with correction for protein oxidation based on 24-h urinary urea nitrogen.

### 2.5. Skeletal Muscle Analysis 

The muscle samples were lyophilized for 48 h and then dissected free of obvious connective tissue, fat, and blood. GS activity was assayed in lyophilized microdissected samples as previously described [14]. Samples were homogenized (0.67% wt/vol soln) in ice-cold buffer (pH 7.5) containing 0.1% 2-mercaptoethanol and (in mmol/L) 10 EDTA, 100 NaF and 0.5 PMSF. The homogenate was centrifuged at 10,000× *g* for 2 min at 4 °C. Total (10 mmol/L G6P) and independent (0–1 mmol/L G6P) GS activity were measured by adding 10 µL of supernatant to 60 µL of reaction mixture containing (in mmol/L) 50 Tris, 20 EDTA, 87.5 KF, 0.2 UDPG, 5000 dpm/nmol (UDP)-[U-14C] glucose, and 1% glycogen. The fractional activity of GS is the independent activity of GS divided by the total activity of GS and is expressed as a percent.

### 2.6. Statistical Analyses

Descriptive statistics were analyzed using SPSS (PASW Statistics, Version 22, Chicago, IL, USA). Differences between stroke and nonstroke control counterparts for physiological characteristics and metabolic variables were determined using unpaired Student’s *t*-tests and variances compared using Levene’s test for equality of variances. GS data were analyzed using SigmaXL v8.15. Extreme outliers (Boxplot rules, <Q1-[3*IQR] or >Q3+[3*IQR]) were identified and removed before further analysis. Paired t-tests were used to compare GS activity between nonparetic and paretic samples in the muscle from stroke survivors. GS activity in control samples was compared to GS activity in nonparetic and paretic muscle samples using classical ANOVA and Fisher’s post-hoc test when there was equal variance, or Welch’s AVOVA, and Welch’s pairwise probability post-hoc test when the assumption of equal variances was violated (Levene’s test for equal variance). Relationships between variables were determined by linear regression analyses with calculation of Pearson product moment correlation coefficients. Data are presented as mean ± SD. Statistical significance was set at a two-tailed *p* < 0.05.

## 3. Results

### 3.1. Physical Characteristics

Subjects with stroke were 60% male and 40% female. The stroke group was racially mixed with 43.3% Caucasian (*n* = 16), 53.3% African-American (*n* = 13), and the remaining 3.3% (*n* = 1) other nationalities. Latency since stroke averaged 4.2 ± 6.9 years (range 0.75–31 years). Approximately 33% of participants with stroke used a single point cane, 23% used a quad cane, 10% used a walker or wheelchair, and 34% did not use any assistive device for ambulation and 37% used an AFO. Controls were 49% male, 51% female and 68.6% Caucasian (*n* = 24) and 28.6% African-American (*n* = 10) and 2.8% (*n* = 1) unidentified. There were no differences in age or BMI between groups (Table 1). Controls had higher fitness (VO_2_max) levels than the stroke group (*p* < 0.005) and higher % body fat (*p* < 0.05). There were no differences in total body fat mass or lean mass by DXA between stroke and control groups. Likewise, central obesity did not differ in terms of visceral fat, subcutaneous abdominal fat, and sagittal diameter at both L_2_-L_3_ and L_4_-L_5_. Mid-thigh muscle area, IMAT area, and muscle attenuation were lower in the paretic than the nonparetic leg (all *p* < 0.005) but expressed relative to the muscle area, %IMAT was higher in paretic than nonparetic (*p* < 0.005). Subcutaneous mid-thigh fat did not differ between paretic and nonparetic legs. The controls have higher mid-thigh muscle area than the muscle area of the paretic limb (*p* < 0.05). 

### 3.2. Glucose Metabolism

There were no differences in fasting glucose or G120 (glucose at 120 min of the OGTT) between stroke and control groups. However, 11 of the stroke participants had known type 2 diabetes and were currently being treated (seven on metformin, glyburide or both; three on some form of insulin injection; one on a DPP4 inhibitor). These individuals did not undergo the OGTT and four stroke participants with known type 2 diabetes mellitus (T2DM) did not undergo a clamp. Two stroke participants and two controls had type 2 diabetes by OGTT (unknown prior to study entry and not on medications). In the stroke group, nine had impaired glucose tolerance (IGT) and 11 controls had IGT. There was no difference in fasting insulin, insulin area under the curve (AUC), glucose AUC, M, and oxidative metabolism during the clamp between stroke and control groups with a trend toward higher glucose storage in controls (Table 2) (*p* = 0.18).

### 3.3. Skeletal Muscle Glycogen Synthase Activity in Stroke Survivors (Paired-t)

Basal activities of GS (independent, total, fractional) were similar between paretic and nonparetic muscle (Table 3). Insulin-stimulated activities of GS (independent, *p* < 0.00005; total, *p* < 3 × 10^−9^; fractional, *p* < 0.05), as well as absolute differences (insulin minus basal) between insulin and basal GS activities (independent, *p* < 0.00005; total, *p* < 5 × 10^−6^; fractional, *p* < 0.05), and the percent change (insulin minus basal, relative to basal) in GS activities (independent, *p* < 0.00001; total, *p* < 1 × 10^−7^; fractional, *p* < 0.05), were all significantly lower in paretic versus nonparetic muscle.

Insulin increased GS independent (*p* < 5 × 10^−6^) and fractional activities (*p* < 5 × 10^−8^) in the nonparetic muscle. Insulin increased GS fractional activity (*p* < 5 × 10^−7^) and decreased GS total activity in the paretic muscle (*p* < 5 × 10^−8^).

### 3.4. Skeletal Muscle Glycogen Synthase Activity in Stroke Survivors vs. Control (ANOVA)

**Figure 1:**
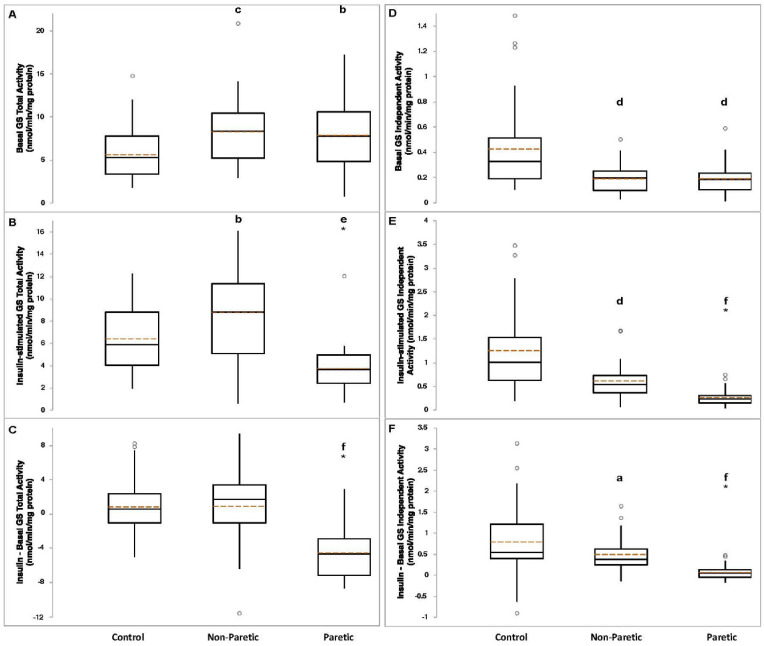
Basal panel (**A**), insulin-stimulated panel (**B**), and absolute change (insulin-stimulated minus basal, panel (**C**) glycogen synthase (GS) independent activities in control (*n* = 34–35), nonparetic (*n* = 24–28), and paretic (*n* = 26–29) muscle samples). Basal panel (**D**), insulin-stimulated panel (**E**), and absolute change panel (**F**) GS total activities in control (*n* = 33–35), nonparetic (*n* = 26–28), and paretic (*n* = 27–29) muscle samples. Boxplot legend: height of box = interquartile range (IQR); top of box = the 75th percentile (Q3); bottom of box = the 25th percentile (Q1); solid center line = median; dashed red line = mean; vertical line = range of data; open circles = outliers (<Q1-[1.5*IQR] or >Q3+[1.5*IQR]). Classical ANOVA with Fisher pairwise probabilities panels (**A**,**C**); Welch’s ANOVA with Welsh’s pairwise probabilities panels (**B**,**D**–**F**). a, *p* < 0.05; b, *p* = 0.01; c, *p* < 0.005; d, *p* < 0.0005; e, *p* < 0.0001; f, *p* < 0.00001 compared to control. * significantly different from nonparetic, *p* < 0.0005.

**Figure 2:**
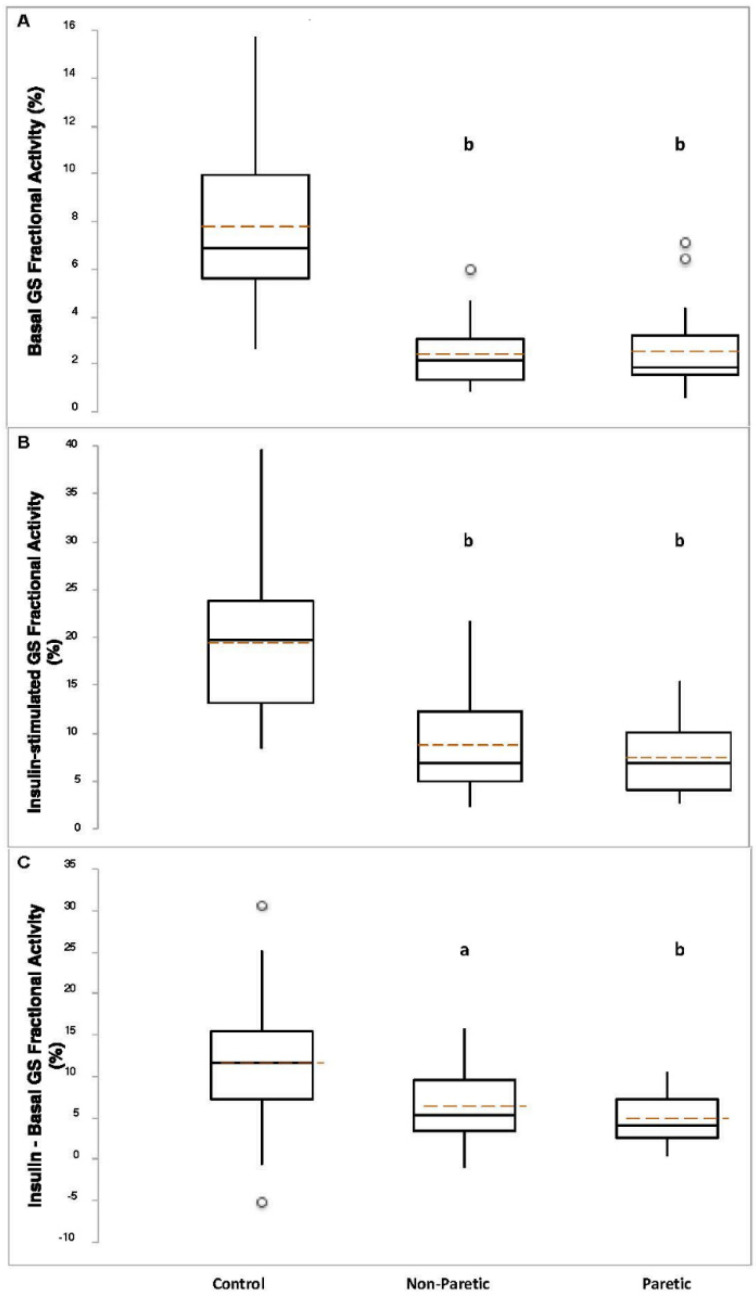
Basal panel (**A**), insulin-stimulated panel (**B**), and absolute change panel (**C**) GS fractional activities in control (*n* = 28–35), nonparetic (*n* = 26–35), and paretic (*n* = 26–35) muscle samples. Boxplot legend: height of box = interquartile range (IQR); top of box = the 75th percentile (Q3); bottom of box = the 25th percentile (Q1); solid center line = median; dashed red line = mean; vertical line = range of data; open circles = outliers (<Q1-[1.5*IQR] or >Q3+[1.5*IQR]). Welch’s ANOVA with Welsh’s pairwise probabilities. a, *p* < 0.0005; b, *p* < 0.00001 compared to control.

Basal GS total activity was lower in control compared to nonparetic (*p* < 0.005) and paretic (*p* = 0.01) muscle samples (Figure 1A). Insulin-stimulated GS total activity was higher in nonparetic (*p* = 0.01) versus control, and lower in paretic (*p* < 0.0001) muscle samples versus control (Figure 1B). The change (insulin minus basal) in GS total activity was lower in paretic muscle samples compared to control (*p* < 3 × 10^−8^, Figure 1C). Basal (*p* < 0.0005), insulin-stimulated (*p* < 0.0005), and the change in GS independent activity (*p* < 0.05) were lower in nonparetic muscle samples compared to control (Figure 1D–F). Basal (*p* < 0.0005), insulin-stimulated (*p* < 3 × 10^−8^), and the change in GS independent activity (*p* < 0.00001) were lower in paretic muscle samples compared to control (Figure 1D–F). Basal (*p* < 2 × 10^−11^) and insulin-stimulated (*p* < 3 × 10^−9^) GS fractional activities, and the change in GS fractional activity (*p* < 0.0005) were lower in nonparetic muscle samples compared to control (Figure 2A–C). Basal (*p* < 4 × 10^−11^) and insulin-stimulated (*p* < 8 × 10^−12^) GS fractional activities, and the change in GS fractional activity (*p* < 0.00001) were lower in paretic muscle samples compared to control (Figure 2A–C).

### 3.5. Relationships between Glucose Metabolism and Body Fat and Fitness

**Figure 3:**
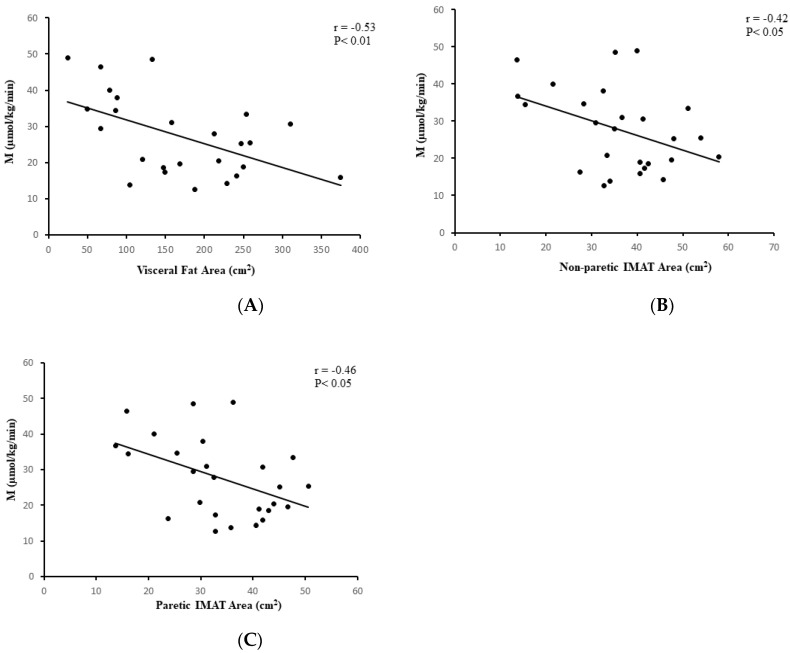
Correlation analysis with body composition. (**A**) Relationship between visceral fat area and glucose utilization (M) in stroke participants (*n* = 25; *r* = −0.53, *p* < 0.01); (**B**) Relationship between nonparetic intermuscular fat (IMAT) area and glucose utilization (M) in stroke participants (*n* = 26; *r* = −0.42, *p* < 0.05); (**C**) Relationship between paretic intermuscular (IMAT) fat area and glucose utilization (M) in stroke participants (*n* = 26; *r* = −0.46, *p* < 0.05).

**Figure 4:**
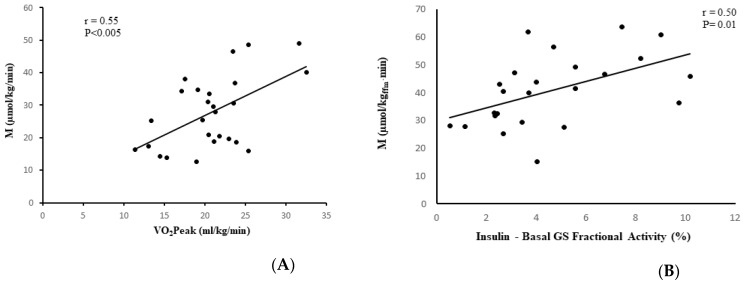
Correlation analysis with fitness and GS. (**A**) Relationship between VO_2_peak and glucose utilization (M) in stroke participants (*n* = 26; *r* = 0.55, *p* < 0.005). (**B**) Relationship between glucose utilization (M) and absolute change in GS fractional activity in paretic muscle in stroke participants (*n* = 24; *r* = 0.50, *p* = 0.01).

In the stroke group, visceral fat at L_4_-L_5_ (*r* = −0.53, *p* < 0.01, *n* = 25, Figure 3A), subcutaneous abdominal fat (*r* = −0.62, *p* < 0.005, *n* = 25), and sagittal diameter (*r* = −0.61, *p* < 0.005, *n* = 25) were associated with lower M. IMAT of the nonparetic leg and paretic leg were also associated with lower M (*r* = −0.42, *p* < 0.05, *n* = 26 and *r* = −0.46, *p* < 0.05, *n* = 26, respectively, Figure 3B,C). VO_2_peak was directly associated with M (r = 0.55, *p* < 0.005, *n* = 26, Figure 4A). Similar relationships were observed in the total group (stroke + controls) for visceral fat at L_4_-L_5_ (*r* = −0.41, *p* < 0.005, *n* = 58), subcutaneous abdominal fat (*r* = −0.35, *p* < 0.01, *n* = 58), and sagittal diameter at L_4_-L_5_ (*r* = −0.47, *p* < 0.0001, *n* = 58) and reduced M.

### 3.6. Skeletal Muscle Glycogen Synthase Activity versus Insulin Sensitivity

M was related to the absolute change in GS fractional activity (insulin-stimulated minus basal) in the control samples (*r* = 0.38, *p* = 0.03, *n* = 33) and in the paretic samples (*r* = 0.50, *p* = 0.01, *n* = 24) (Figure 4B), but not in the nonparetic samples (*r* = 0.25, *p* = 0.24, *n* = 24).

## 4. Discussion

The present study is the first to demonstrate reduced insulin activation of glycogen synthase in the paretic limb compared to the nonparetic limb in stroke participants. Compared to nonstroke controls, stroke survivors had lower insulin-stimulated independent and fractional activity. We also present the first evidence of the relationships between fitness, abdominal fat, and intermuscular fat with glucose utilization determined by a glucose clamp in stroke survivors.

Diabetes is an independent and modifiable risk factor for stroke with mechanisms including systemic inflammation, endothelial dysfunction, increased early-age arterial stiffness, and thickening of the capillary basement membrane [20]. There are only two studies in the literature that have measured insulin sensitivity by the glucose clamp in chronic stroke participants including one of our own where we showed that resistive training improved insulin action by the hyperglycemic clamp in chronic stroke [21]. In the second study, an increase in the number of silent cerebral infarcts correlated with glucose uptake during a hyperinsulinemic-euglycemic clamp in a small number of patients with hypertension [22]. Additionally, in hypertensive patients with cerebral infarction, inflammation (hs-CRP and IL-6 levels) and insulin resistance by homeostatic model assessment insulin resistance (HOMA-IR) index were associated with the diameter of the cerebral infarct [23]. In nondiabetic subjects with ischemic stroke, HOMA-IR was associated with a worse functional outcome measured by the modified Rankin scale [24]. Although we hypothesized that the stroke group would be more insulin resistant by the glucose clamp, the lower M and 15% lower nonoxidative metabolism in the stroke group was not significantly different than controls. Since about one-third of the stroke participants were being treated for T2DM, this could have influenced these comparisons. 

Aerobic capacity is a significant contributor to insulin sensitivity [25,26] and changes in VO_2_max with exercise training predict improvements in insulin sensitivity [14]. We demonstrate, for the first time, a direct relationship between VO_2_peak and M in stroke survivors suggesting that even in disabled individuals, fitness is an important indicator of insulin sensitivity. We [25] and others [8] have reported the relationships of visceral and abdominal fat with insulin resistance and provide new evidence that this association is observed in stroke survivors. Likewise, increased IMAT is associated with reduced insulin sensitivity in obesity and diabetes [8]. Our current data implicate IMAT in paretic and nonparetic skeletal muscle in insulin resistance by the glucose clamp and support our earlier finding of the relationship between higher IMAT and higher fasting insulin concentrations in chronic stroke [4]. In human primary muscle cells incubated with conditioned media from IMAT, the IMAT expression of genes in insulin signaling including the JAKSTAT and MAPK signaling pathways, oxidative phosphorylation, inflammatory cytokine gene expression (e.g., plasminogen activator inhibitor 1, monocyte chemotactic protein 1, TNF alpha-induced protein 3 among others), and peroxisomal metabolism related to insulin sensitivity of the donor indicating that numerous factors secreted from IMAT may regulate insulin resistance [27]. Given our earlier findings of paretic skeletal muscle inflammation and increased IMAT in paretic muscle [4,5,10], it is compelling to suggest insulin sensitivity is negatively influenced by inflammation in stroke survivors through these pathways but that remains to be explored. Adipokines may also be contributing factors as our earlier work demonstrated significantly lower circulating adiponectin levels in stroke survivors with diabetes, than those with impaired glucose tolerance and highest levels observed in stroke patients with normal glucose tolerance [28].

Our new findings indicate that basal GS independent activity is 40% lower in stroke muscle compared to control muscle. Independent (independent on the allosteric activator glucose-6-phosphate) activity reflects the active, dephosphorylated form of GS. Surprisingly, basal GS total activity was almost 150% higher in stroke muscle compared to control. The higher GS total activity may reflect a compensatory mechanism to overcome the lower independent activity of GS in the stroke muscle. GS total activity has been found to be lower in muscle and cultured cells of individuals with insulin resistance and type 2 diabetes compared to controls [29,30,31,32]. In the current study, lower independent and higher total GS activity in the stroke muscle resulted in control muscle having a remarkable 3-fold higher fractional activity (independent relative to total) under basal conditions.

A unique feature of our investigation was the determination of GS in both paretic and nonparetic skeletal muscle which eliminates genetic variation, with the nonparetic muscle serving as an internal control in the stroke group and comparison to nonstroke control muscle. There is abnormal neural activation in the paretic limb which results in reduced muscle size and strength [4,33] and disuse; factors which could influence metabolism. During insulin-stimulation, GS independent activity in paretic muscle was 40% lower than nonparetic and 80% lower than control muscle. The absolute change in GS independent activity was lower in paretic muscle compared to nonparetic and control muscle. It is possible that in stroke muscle, an essential defect in GS causes it to be locked in the inactive phosphorylated form, the paretic muscle to a greater degree than the nonparetic muscle (GS has nine phosphorylation sites), as has been demonstrated in individuals with type 2 diabetes [34]. During the hyperinsulinemic-euglycemic clamp, total GS activity was higher in the nonparetic compared to control and paretic muscle, yet the absolute change in total GS activity in nonparetic muscle was similar to the effect of insulin in the control muscle. Paretic muscle had 50–67% less insulin-stimulated GS total activity, and lower absolute change in total activity compared to control and paretic muscle. Insulin-stimulated GS fractional activity was 2.5-fold higher in the control samples, and the absolute changes in GS fractional activity was 2-fold higher in the control samples versus stroke muscle samples. Reduced insulin-activation of skeletal muscle GS during a hyperinsulinemic-euglycemic clamp is a hallmark of insulin resistance and type 2 diabetes.

Whereas insulin resistance and type 2 diabetes are associated with lower insulin-mediated whole-body glucose disposal, which is in part due to lower insulin activation of skeletal muscle GS [14,35], the stroke survivors did not have lower insulin-mediated glucose disposal compared to controls in the current study. Although we did not measure GLUT4 in the current study, one explanation for similar glucose disposal during the hyperinsulinemic clamp in the control and stroke survivors could be that GLUT4 expression or GLUT4 translocation was similar or greater in the stroke survivors which could serve to overcome the lower insulin-mediated glycogen synthase activity (i.e., “push” greater than “pull” mechanism [36]). In the current study, the change in GS fractional activity during the clamp was related to M in the controls as expected. Of note, the change in GS fractional activity in the nonparetic muscle was not related to M, whereas the change in GS fractional activity during the clamp in the paretic samples was related to M, creating different contributions of affected and nonaffected stroke muscle to systemic insulin action. 

A limitation of this study is that there were a greater proportion of stroke participants who had treated T2DM than the control cohort which could potentially confound the comparison between these two groups. Other limitations to the data include the inclusion of participants with and without diabetes in each group, lack of data on muscle fiber characteristics, and modest correlations. Yet, both groups had the same number of participants with newly diagnosed, untreated T2DM and similar proportions of IGT. In addition, the groups were well-matched for other confounders such as age and BMI. Moreover, the subjects studied represent a racially and sex mixed group, which is statistically similar to stroke survivors in the U.S. [37]. Given that lifestyle factors such as physical activity, nutrition, smoking, and obesity can influence neurodegenerative diseases and stroke [38], future studies could examine these in relationship to insulin resistance in chronic stroke.

## 5. Conclusions

Our findings indicate that in stroke survivors, insulin activation of glycogen synthase is reduced in the paretic compared to nonparetic muscle and that whole-body insulin resistance was related to reduced fitness, visceral and intramuscular fat, and reduced insulin activation of GS. Stroke survivors show reduced insulin action on skeletal muscle GS independent and fractional activity compared to nonstroke individuals. Our results suggest there is a disconnect between insulin resistance at the muscle (reduced insulin activation of GS) and whole-body insulin resistance in stroke survivors. The mechanisms by which whole body glucose disposal is influenced after stroke requires further study. Future studies could also be directed at interventions such as exercise training to improve insulin sensitivity in stroke survivors as well as include an in-depth examination of the mechanisms in paretic and nonparetic skeletal muscle in this disabled population.

## Figures and Tables

**Table 1:** Physical and metabolic characteristics.

	Stroke (*n* = 30)	Controls (*n* = 35)
Age (yr)	60 ± 8	62 ± 8
Weight (kg)	93.2 ± 21.5	96.2 ± 16.9
BMI (kg/m^2^)	31.6 ± 7.5	33.5 ± 5.0
% Body fat	37.6 ± 8.6	41.8 ± 8.2 *
Fat mass (kg)	36.1 ± 13.8	40.5 ± 10.8
Fat-free mass (kg)	58.3 ± 11.4	55.9 ± 11.6
VO_2_max (mL/kg/min)	20.1 ± 5.1	23.7 ± 5.2 †
VO_2_max (L/min)	1.82 ± 0.55	2.29 ± 0.59 ‡
Visceral fat area L_2_-L_3_ (cm^2^)	226.8 ± 109.7	229.7 ± 109.9
Visceral fat area L_4_-L_5_(cm^2^)	169.7 ± 85.5	182.3 ± 71.4
Subcutaneous abdominal fat L_2_-L_3_ (cm^2^)	308.6 ± 151.6	317.3 ± 121.8
Subcutaneous abdominal fat L_4_-L_5_ (cm^2^)	364.2 ± 177.1	423.6 ± 140.6
Sagittal diameter L_2_-L_3_ (cm)	27.3 ± 5.3	26.8 ± 3.5
Sagittal diameter L_4_-L_5_ (cm)	26.7 ± 5.8	27.6 ± 3.9
Mid-thigh NP muscle area (cm^2^) Mid-thigh NP muscle (%)	91.2 ± 17.1 57.2 ± 1.1	88.0 ± 26.8 **
Mid-thigh P muscle area (cm^2^) Mid-thigh P muscle (%)	74.4 ± 17.2 ‡‡ 52.3 ± 1.0 ‡‡‡	
Mid-thigh NP subcutaneous fat (cm^2^)	119.6 ± 64.9	142.5 ± 71.7
Mid-thigh P subcutaneous fat (cm^2^)	121.9 ± 63.3	
Mid-thigh NP IMAT (cm^2^) Mid-thigh NP IMAT (%)	35.7 ± 11.3 21.4 ± 3.2	34.8 ± 10.4
Mid-thigh P IMAT (cm^2^) Mid-thigh P IMAT (%)	33.3 ± 10.1 ‡‡ 22.6 ± 2.6 ‡‡	
Mid-thigh NP muscle attenuation (HU)	37.8 ± 4.8	36.2 ± 6.9
Mid-thigh P muscle attenuation (HU)	33.8 ± 6.1 ‡‡	

Values are means ± SD. NP = nonparetic; P = paretic. Significantly different between stroke and control: * *p* < 0.05; † *p* < 0.01; ‡ *p* < 0.005. Significantly different between NP and P ‡‡ *p* < 0.005 or ‡‡‡ *p* < 0.0001. Significantly different between *p* and control ** *p* < 0.05.

**Table 2:** Glucose metabolism.

	Stroke (*n* = 30) ^a^	Controls (*n* = 35) ^a^
Fasting plasma glucose (mmol/L)	5.40 ± 0.99	5.46 ± 0.66
Fasting plasma insulin (pmol/L)	105 ± 80	106 ± 49
Glucose at 120 min	7.74 ± 2.17	7.90 ± 2.19
Insulin at 120 min	622 ± 552	661 ± 397
Glucose AUC (mmol/L·120 min)	1014 ± 202	989 ± 213
Insulin AUC (pmol/L·120 min)	68,928 ± 58,589	68,823 ± 34,992
M (µmol·kg^−1^·min^−1^)	27.7 ± 11.0	27.2 ± 10.5
M (µmol·kg_FFM_^−1^·min^−1^)	42.1 ± 15.9	45.7 ± 18.1
CHO oxidative metabolism (µmol·kg_FFM_^−1^·min^−1^)	6.67 ± 7.05	7.91 ± 8.41
Nonoxidative metabolism (µmol·kg_FFM_^−1^·min^−1^)	35.98 ± 14.96	41.94 ± 18.30

Values are means ± SD. AUC = area under the curve; M = glucose utilization; CHO = carbohydrate. ^a^ unless denoted differently in the manuscript.

**Table 3:** Glycogen synthase activity in stroke survivors (*n* = 23) before (basal) and during (insulin) a euglycemic clamp.

Glycogen Synthase Activity			
	Nonparetic	Paretic	*p*-Value
Basal GSI (nmol/min/mg protein)	0.19 ± 0.12	0.20 ± 0.13	0.80
Insulin GSI (nmol/min/mg protein)	0.66 ± 0.39 *	0.21 ± 0.13	0.00003
Insulin-Basal GSI (nmol/min/mg protein)	0.53 ± 0.42	0.008 ± 0.12	0.00004
% above Basal GSI	413 ± 338	18 ± 65	0.000009
Basal GST (nmol/min/mg protein)	8.5 ± 4.1	7.9 ± 3.3	0.43
Insulin GST (nmol/min/mg protein)	9.5 ± 3.6	3.3 ± 1.5 ‡	0.000000003
Insulin-Basal GST (nmol/min/mg protein)	1.0 ± 4.3	−4.8 ± 2.6	0.000004
% above Basal GST	24 ± 47	−61 ± 14	0.0000001
Basal GSFV (%)	2.4 ± 1.4	2.6 ± 1.6	0.51
Insulin GSFV (%)	8.6 ± 5.1 ‡	6.9 ± 3.5 †	0.048
Insulin-Basal GSFV (%)	6.2 ± 4.3	4.3 ± 2.5	0.02
% above Basal GSFV	296 ± 156	205 ± 134	0.02

Data are presented as (mean ± SD). basal vs. insulin, paired-t test, * *p* < 0.000005; † *p* < 0.0000005; ‡ *p* < 0.00000005 vs. basal.
